# Clinical Significance of Survivin Expression in Patients with Urothelial Carcinoma

**DOI:** 10.1155/2014/574985

**Published:** 2014-02-05

**Authors:** Hsin-An Chen, Chih-Ming Su, Hsiao-Yen Hsieh, Chun-Liang Tung, Cheng-Da Hsu, Yuan-Hung Wang, Cheng-Huang Shen

**Affiliations:** ^1^Division of General Surgery, Department of Surgery, Shuang Ho Hospital, Taipei Medical University, New Taipei City 23561, Taiwan; ^2^Graduate Institute of Clinical Medicine, College of Medicine, Taipei Medical University, 250 Wu-Hsing Street, Taipei 110, Taiwan; ^3^Department of Biomedical Research, Chiayi Christian Hospital, Chiayi 600, Taiwan; ^4^Department of Pathology, Chiayi Christian Hospital, Chiayi 600, Taiwan; ^5^Division of General Surgery, Department of Urology, Shuang Ho Hospital, Taipei Medical University, New Taipei City 23561, Taiwan; ^6^Department of Urology, Chiayi Christian Hospital, 539 Chung Hsiao Road, Chiayi 600, Taiwan; ^7^Tainan University of Technology, Tainan City 71002, Taiwan

## Abstract

*Background*. Survivin is a member of the inhibitors of apoptosis protein family that plays an important role in carcinogenesis. Here, we examined the association between survivin expression and clinical outcome in urothelial carcinoma of the bladder (UCB). *Methods*. A total of 56 histopathologically confirmed UCB patients were recruited from the Department of Urology of Chiayi Christian Hospital from August 2007 to May 2009. Immunohistochemistry (IHC) was used to detect the survivin expression in tumor tissues. The –31 C/G polymorphism in *survivin* promoter region was determined by polymerase chain reaction-restricted fragment length polymorphism. *Results*. The frequency of high survivin expression was significantly higher in muscle-invasive tumors (66.6%) than in non-muscle-invasive tumors (34.2%) (*P* = 0.042) and in poorly differentiated (85.7%) tumors than in moderately differentiated tumors (30.8%) (*P* = 0.0014). The higher frequency of risk genotypes (C/C and C/G) was found in the median (72.7%) and high (68.0%) survivin expression groups. The multivariate analysis showed that a high survivin expression level was a potential predictive biomarker of poor overall survival (*P* = 0.02). *Conclusion*. Our results suggest that the high survivin expression was associated with tumor stage and grade and may present a predictive marker of overall survival in UCB.

## 1. Introduction

Urothelial carcinomas (UCs) arise from the urothelium of the urinary tract and include cancers of the bladder, renal pelvis, and ureter. According to the 2012 annual report of the Taiwan Cancer Registry, the age-standardized incidence rate of bladder cancer per 100,000 persons was 9.42 in males and 3.66 in females [1]. UCs are the second most common malignancy of the genitourinary tract worldwide and UC of the bladder (UCB) is the ninth most common malignancy among men in Taiwan, where it accounts for approximately 1000 deaths annually [2]. Twenty percent UCB patients have muscle-invasive or metastatic tumors at the first presentation and half of these patients die within 2-3 years. In addition, 75%–80% UCB patients present with superficial tumors and, of these, 30%–85% experience recurrence and 10%–30% progress to muscle-invasive tumors with advanced stages, grades, and poor prognoses [3, 4]. In recent years, advances in biomedical technologies have led to the discovery of novel biomarkers to predict clinical diagnosis, prognosis, and individual susceptibility to treatment. Although the detailed mechanisms and applications of these biomarkers to effectively improve clinical outcomes of various malignancies remain unknown, the discovery of additional potential molecular biomarkers will further aid in the clinical diagnosis, treatment, and prognosis of UCB.

Apoptosis is a critical mechanism in the regulation of cell growth, division, and death. Survivin, or baculoviral inhibitor of apoptosis repeat-containing 5, is a member of the inhibitors of apoptosis protein (IAP) family [5, 6]. Survivin is expressed during the G2/M phase of the cell cycle and directly inhibits caspase-3 and caspase-7 activity [7, 8]. The molecular structure of survivin has only one N-terminal baculovirus IAP repeat domain and a long C-terminal helix-coiled region [9]. A characteristic of the promoter region of *survivin* is the existence of a cell cycle-dependent element (CDE) and a cell cycle homology region (CHR) [10]. *Survivin* gene is located at chromosome 17q25 and encodes the 16.3 kDa, 142-amino acid survivin protein. Several single-nucleotide polymorphisms (SNPs) have been identified in the promoter region of *survivin* [6, 11]. In our previous report, we identified a –31 C/G polymorphism located within the CDE/CHR repressor binding site, which was found to be associated with tumor stage and grade in UCs [12].

Several previous studies have reported that survivin overexpression was significantly associated with various malignancies, such as cancers of the bladder, prostate, colorectum, and lung [13–17]. In addition, some immunohistochemistry (IHC) studies reported survivin expression in a high proportion of UC patients [18, 19]. In another study, survivin expression was observed in tumor cells, but not in normal urothelial cells, in patients with superficial bladder cancer [20, 21]. However, further studies are required to clarify the practical application of survivin as a useful biomarker of UCB clinical characteristics. Based on the important role of survivin in carcinogenesis, we investigated the association between survivin expression and UCB clinical outcome and also proposed that the –31 C/G polymorphism of *survivin* promoter might modulate its expression, thereby affecting individual susceptibility to UCB development.

## 2. Materials and Methods

### 2.1. Study Subjects and Tissue Samples

In the present study, a total of 56 histologically confirmed UCB patients, who were treated at Chiayi Christian Hospital (Chiayi City, Taiwan) from August 2006 to May 2007, were retrospectively analyzed. Based on the World Health Organization grading system, the histological characteristics of transitional cell carcinoma (TCC) were classified into 3 grades (grades 1–3). Staging of bladder TCC was classified using the tumor node metastasis system into 2 subgroups (stages T1 or T2–T4), whereas the pathological grade was divided into 3 groups (grades G1–G3) as previously described [12, 22]. All subjects received a detailed description of this study and provided written informed consent before inclusion. The institutional review board of Chiayi Christian Hospital approved the study protocol.

### 2.2. Immunohistochemistry (IHC) Analysis

Survivin protein expression in clinical samples was subjected to IHC analysis. In brief, tissue samples were embedded in paraffin blocks, cut into 3 *μ*m sections, deparaffinized, and rehydrated. Next, the sections were mounted on slides, which were immersed in a 10 mM citrate buffer (pH = 6.0), heated in a microwave oven three times for 5 min each, treated with endogenous peroxidase in 1.5% H_2_O_2_ for 20 min, and incubated with rabbit polyclonal anti-survivin antibody (dilution, 1 : 2400; ab469, Abcam Plc., Cambridge, England) for 1 h at room temperature in a humidified chamber. Next, the slides were washed with phosphate-buffered saline three times and one drop of Super Enhance and poly-horseradish peroxidase reagents (BioGenex, San Ramon, CA, USA) was added to cover the specimen, and the slides were incubated for 20 min at room temperature. Color was developed by incubating the slides in substrate solution for 4–8 min at room temperature and then counterstained using Mayer's hematoxylin. A negative control slide (without primary antibody) was included for each staining. The intensity of the reactions was assessed semiquantitatively using three expression categories: 0–5% (low expression), 5%–50% (moderate expression), and >50% (high expression).

### 2.3. Genotyping of *Survivin *–31 C/G Polymorphism

Genomic DNA was extracted from 200 *μ*L of whole blood using conventional proteinase K digestion and the phenol/chloroform extraction method. *Survivin* promoter region polymorphism at –31 C/G was analyzed as previously described [12].

### 2.4. Statistical Analysis

The chi-squared test was used to examine the association between survivin expression and clinicopathological characteristics. Kaplan-Meier survival analysis and the log-rank test were used to assess differences in overall survival (OS) between UCB patients with high and low-to-median survivin expression. Multiple Cox proportional hazard regression analysis was used to estimate the independent prognostic effect of survivin expression after adjustment for patient age and tumor stage and grade. A probability *P* value <0.05 was considered statistically significant. Statistical analysis was performed using SAS software ver. 9.1 (SAS Institute Inc., Cary, NC, USA).

## 3. Results

### 3.1. Basic Characteristics

**Figure 1:**
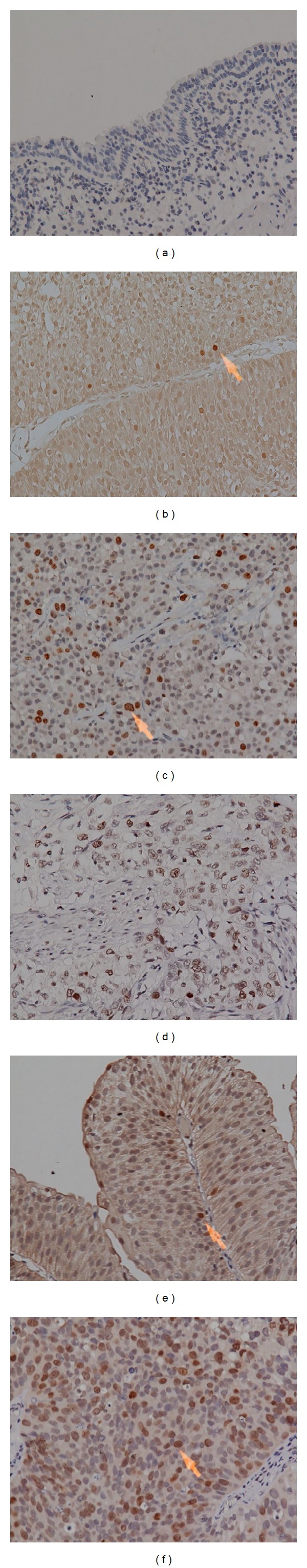
Immunohistochemical staining for survivin in (a) normal urothelium; (b) tumor grade G1; (c) tumor grade G2; (d) tumor grade G3; (e) superficial; (f) invasive UCB from transurethral resection specimens.

Of the 56 UCB patients, the mean and standard deviation (SD) of age was 69.1 ± 12.6 years and 64.3% were male. Regarding the tumor stage, the frequencies of non-muscle- and muscle-invasive tumors were 67.8% and 32.2%, respectively. In terms of tumor grade, the frequencies of G1, G2, and G3 were 23.2%, 51.8%, and 25.0%, respectively. The frequencies of low, moderate, and high survivin expression levels in UCB tumor tissues were 16.1%, 39.3%, and 44.6%, respectively (Table 1). As shown in Figure 1, the IHC-stained cells that were positive for survivin expression were classified as low (0–5%), moderate (6%–50%), or high (>50%) survivin expression.

### 3.2. Survivin Expression and Clinical Characteristics

In non-muscle-invasive UCB patients, the frequencies of low, moderate, and high survivin expression were 15.8%, 50.0%, and 34.2%, respectively, and in muscle-invasive patients, the frequencies were 16.7%, 16.7%, and 66.6%, respectively. The frequency of high survivin expression was significantly higher in muscle-invasive cases (*P* = 0.042) and poorly differentiated tumors (85.7%) compared with moderately differentiated tumors (30.8%) (*P* = 0.0014) (Table 2).

### 3.3. Survivin Expression and the –31 C/G Polymorphism

The distributions of the –31 C/G polymorphism in *survivin* promoter were 26.8%, 39.3%, and 33.9% for the C/C, C/G, and G/G genotypes, respectively. The frequencies of individuals with at least one variant –31 C allele (risk genotypes, C/C and C/G) in the moderate and high survivin expression groups were higher than those in the low expression group. The frequency distribution of the –31 C/G polymorphism differed significantly between the survivin expression groups (*P* = 0.041) (Table 3).

### 3.4. Multivariate Analysis of Disease-Free and Overall Survival

**Figure 2:**
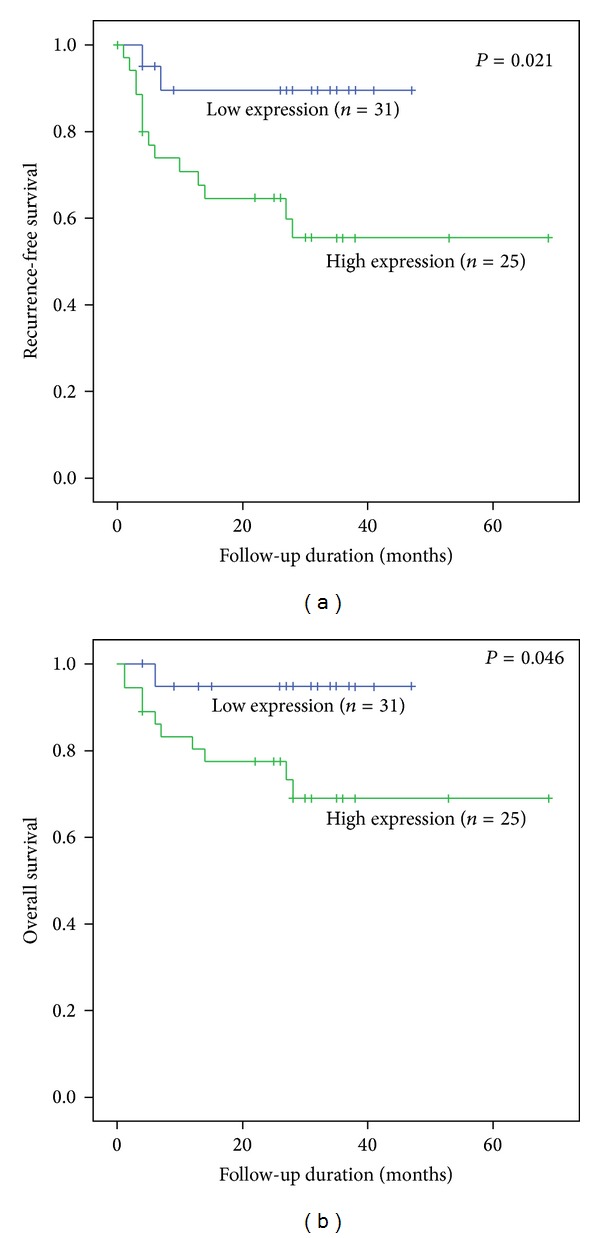
Disease-free survival (a) and overall survival (b) analyses in different survivin expression groups. The Kaplan-Meier method was used to compare the differences between subgroups (high versus low-median) and the significance was determined by log-rank test.

The prognostic effects of high survivin expression on disease-free survival (DFS) and OS of UCB were estimated using the Cox proportional hazard model. We observed significantly poorer DFS and OS rates for UCB patients with high survivin expression (61.1% and 72.2%, resp.) than for those with low survivin expression (90.0% and 95.0%, resp.) (Figure 2). The associations of high survivin expression and DFS and OS rates for all 56 UCB patients are shown in Table 4. Multivariate analysis showed that UCB patients with high survivin expression levels had an increased risk of poorer DFS (hazard ratio, HR = 3.2, *P* = 0.26). Regarding OS, UCB patients with high survivin expression levels had a significantly greater risk of poorer OS (HR = 12.3, *P* = 0.02).

## 4. Discussion

In the present study, the prevalence of high survivin expression was significantly greater in muscle-invasive tumors than in non-muscle-invasive tumors. In addition, the frequency of high survivin expression was significantly greater in poorly differentiated than moderately differentiated tumors. More importantly, high survivin expression levels were significantly associated with OS in UCB. These findings suggested that a high survivin expression level was a potential predictive biomarker of UBC prognosis.

Survivin is a member of the IAP family and is involved in triggering of tumor cell apoptosis, dysregulation of mitotic progression, and inhibition of tumor growth [23]. Dysregulation of the survivin pathway may participate in early initiation of malignant transformation and later maintenance of the malignant phenotype of established tumors [24]. *Survivin* is located on chromosome 17q25 and encodes a 142-amino acid protein. More than 10 common SNPs have been identified in the *survivin* promoter region, in which the –31 C/G polymorphism is one of the most common variants [25, 26]. To date, several epidemiological studies have suggested that the –31 C/G polymorphism was associated with the risk and/or prognosis of various carcinomas [6]. In our previous study, we found that the –31 C/G polymorphism was associated with tumor stage and grade in UCs, which suggested an important role of the SNP in the development of urinary system cancer [12].

In the present study, we observed that the *survivin*  –31 CC genotype was associated with high survivin expression levels. This finding suggested that the mutant polymorphism up-regulated cell-cycle-dependent survivin transcription and resulted in overexpression at both the mRNA and protein levels [15, 27, 28]. Furthermore, our results showed that survivin overexpression was significantly correlated with the advanced tumor stage and grade. In a multivariate analysis (Table 4), we identified that survivin overexpression was a significant predictor of OS. In addition, we also found that UCB patients with survivin overexpression had a nonsignificantly increased risk of DFS (HR = 3.2). Due to the smaller number of UCB patients with recurrence, the effect of survivin overexpression on DFS had a lower statistical power.

To date, survivin expression has been reported as a novel prognostic factor in several human malignancies and survivin expression in tumors was correlated to more aggressive behavior and poor prognosis [29–33]. Taken together, the results of our present study suggested that a high survivin expression level was a potential predictive biomarker of UCB progression and poor prognosis. When interpreting our results, some limitations should be addressed. The sample size in the present study was relatively small and may not provide sufficient statistical significance to estimate the correlation between high survivin expression and OS. In addition, we only investigated 1 SNP of the *survivin* gene, which might not sufficiently account for gene expression. Further studies investigating more SNPs of the *survivin* gene with a larger sample size are necessary to validate our findings.

In conclusion, our results indicated that a high survivin expression level was associated with tumor stage and grade and may be a predictive marker of OS in UBC. Although these associations appeared to be statistically significant in a Chinese population, our initial OS and DFS findings should be prospectively confirmed in a larger cohort of UBC patients.

## Figures and Tables

**Table 1:** Basic characteristics of 56 patients with UCB.

	56 patients with UCB *N* (%)
Age (years)	
<55	18 (32.2)
55–69	12 (21.4)
≥70	26 (46.4)
Mean ± SD	69.1 ± 12.6
Gender	
Female	20 (35.7)
Male	36 (64.3)
Tumor stage	
Non-muscle-invasive (Ta-T1)	38 (67.8)
Muscle-invasive (T2–T4)	18 (32.2)
Tumor grade	
G1	13 (23.2)
G2	29 (51.8)
G3	14 (25.0)
IHC expression^a^	
Low	9 (16.1)
Moderate	22 (39.3)
High	25 (44.6)

^a^Percentage of survivin (+) cells: low, 0–5%; moderate, 5–50%; high, >50%.

**Table 2:** Relationship between IHC expression of survivin and clinical characteristics.

	Survivin expression in tumor tissues^a^			*P* value^b^
	Low	Moderate	High	
Tumor stage				
Non-muscle-invasive (Ta-T1)	6 (15.8)	19 (50.0)	13 (34.2)	0.042*
Muscle-invasive (T2–T4)	3 (16.7)	3 (16.7)	12 (66.6)	
Tumor grade				
G1	4 (30.8)	5 (38.4)	4 (30.8)	0.0014**
G2	3 (10.4)	17 (58.6)	9 (31.0)	
G3	2 (14.3)	0 (0.0)	12 (85.7)	

*P < 0.05; **P < 0.01. ^a^Percentage of survivin (+) cells: low, 0–5%; moderate, 5–50%; high, >50%. ^b^Chi-square test.

**Table 3:** Relationship between survivin expression and the –31 G/C polymorphism.

	Expression level in tumor tissues^a^			*P* value^b^
	Low *n* (%)	Moderate *n* (%)	High *n* (%)	
–31 C/G polymorphism				
G/G	5 (55.6)	6 (27.3)	8 (32.0)	0.041*
C/C + C/G	4 (44.4)	16 (72.7)	17 (68.0)	
Total	** 9 (100.0)**	** 22 (100.0)**	** 25 (100.0)**	

*P < 0.05. ^a^Percentage of survivin (+) cells: low, 0–5%; moderate, 5–50%; high, >50%. ^b^Chi-square test.

**Table 4:** Multivariate analysis of DFS and OS of 56 patients with UCB.

Variables	HR	95% CI	*P* value
Disease-free survival (DFS)			
Age (≥65 versus <65 years)	1.7	0.6–4.8	0.29
Tumor stage (MI versus NMI)^a^	1.6	0.6–4.4	0.38
Tumor grade (G2-3 versus G1)	1.5	0.5–9.8	0.49
Survivin expression (high versus low)	3.2	0.4–24.5	0.26
Overall survival (OS)			
Age (≥65 versus <65 years)	1.8	0.8–17.6	0.08
Tumor stage (MI versus NMI)^a^	2.9	0.8–9.9	0.10
Tumor grade (G2-3 versus G1)	2.1	0.6–10.1	0.46
Survivin expression (high versus low)	12.3	1.5–98.4	0.02

^a^MI: muscle-invasive; NMI: non-muscle-invasive.

## Abbreviations

UCB: Urothelial carcinoma of the bladder
IHC: Immunohistochemistry
DFS: Disease-free survival
OS: Overall survival.
